# Supplementary material for: Seasonality and Trends in Stevens-Johnson Syndrome/Toxic Epidermal Necrolysis Before and During the COVID-19 Pandemic: A Pharmacovigilance Study
Source: medRxiv. 2025 Aug 24:2025.08.20.25331677. Preprint. [Version 1] doi: 10.1101/2025.08.20.25331677 (PMC12393647; doi:10.1101/2025.08.20.25331677)
Supplement: 2 [file NIHPP2025.08.20.25331677v1-supplement-2.pdf]

medRxiv preprint doi: https://doi.org/10.1101/2025.08.20.25331677; this version posted August 24, 2025. The copyright holder for this preprint (which was not certified by peer review) is the author/funder, who has granted medRxiv a license to display the preprint in perpetuity.
It is made available under a CC-BY 4.0 International license .

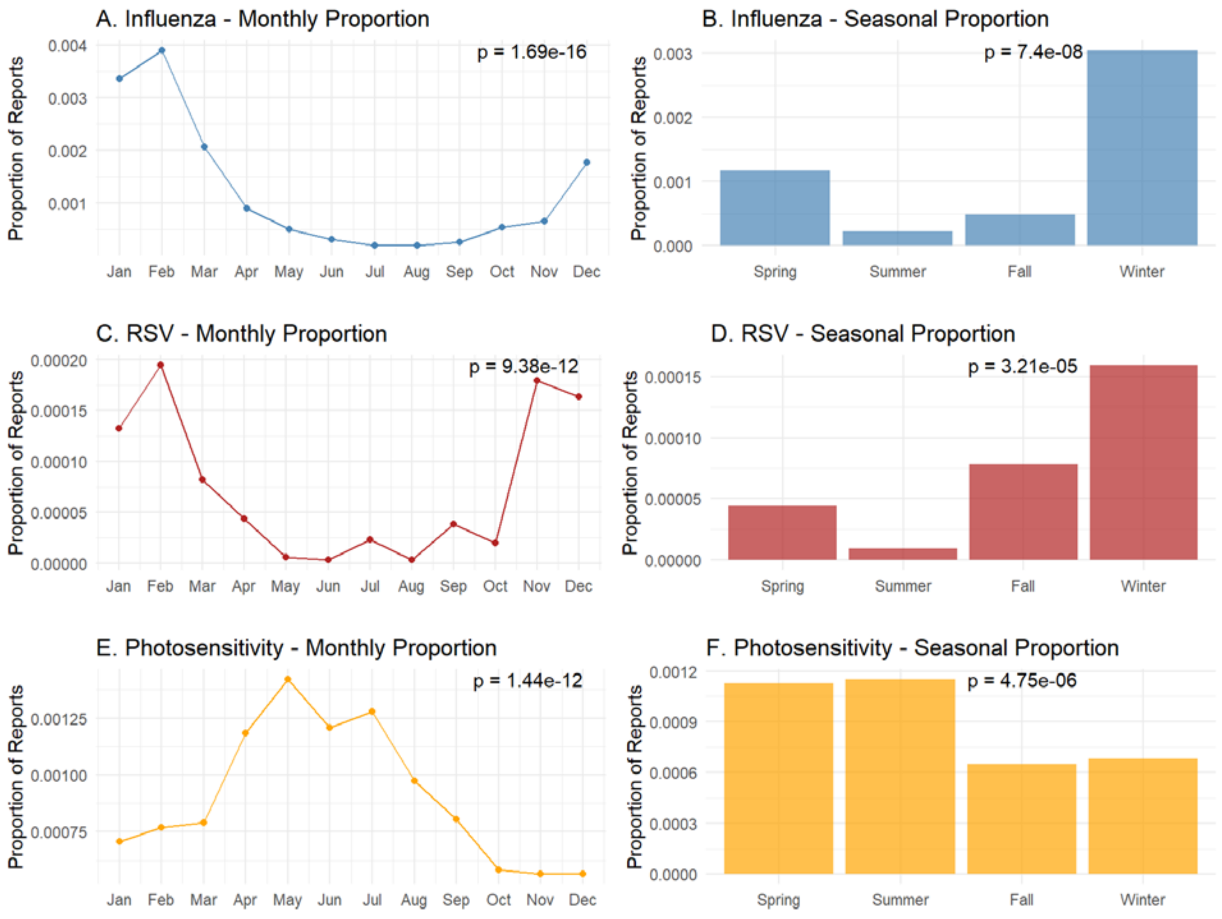

eFigure 1. Monthly and Seasonal Patterns in Photosensitivity and Respiratory Virus Reports. Monthly and seasonal proportions of photosensitivity, influenza, and RSV reports submitted to FAERS from 2010-2019 are shown. Proportions represent the number of unique reports for each outcome relative to all FAERS reports submitted during each time period. Seasons are defined using Northern Hemisphere convention: Winter (December-February), Spring (March-May), Summer (June-August), and Fall (September-November). Kruskal-Wallis tests for both monthly and seasonal variation were significant for all three outcomes (p-values shown on each panel).

medRxiv preprint doi: https://doi.org/10.1101/2025.08.20.25331677; this version posted August 24, 2025. The copyright holder for this preprint (which was not certified by peer review) is the author/funder, who has granted medRxiv a license to display the preprint in perpetuity.
It is made available under a CC-BY 4.0 International license .

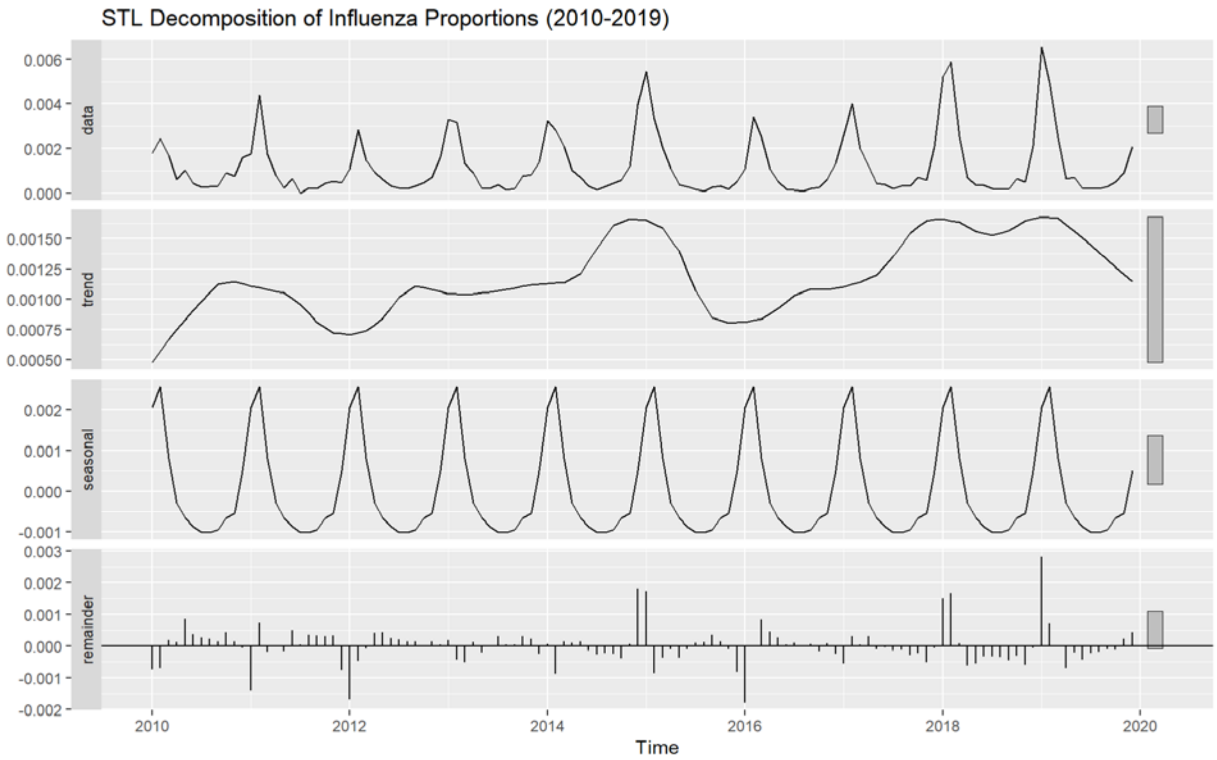

eFigure 2. STL Decomposition of Influenza Report Proportions Over Time. The raw time series (top panel) was decomposed into a long-term trend component (second panel), a seasonal component reflecting recurring within-year fluctuations (third panel), and a remainder capturing unexplained variation (bottom panel). Strong seasonal structure is observed, consistent with a high seasonality strength of 0.8 calculated from this decomposition.

medRxiv preprint doi: https://doi.org/10.1101/2025.08.20.25331677; this version posted August 24, 2025. The copyright holder for this preprint (which was not certified by peer review) is the author/funder, who has granted medRxiv a license to display the preprint in perpetuity.
It is made available under a CC-BY 4.0 International license .

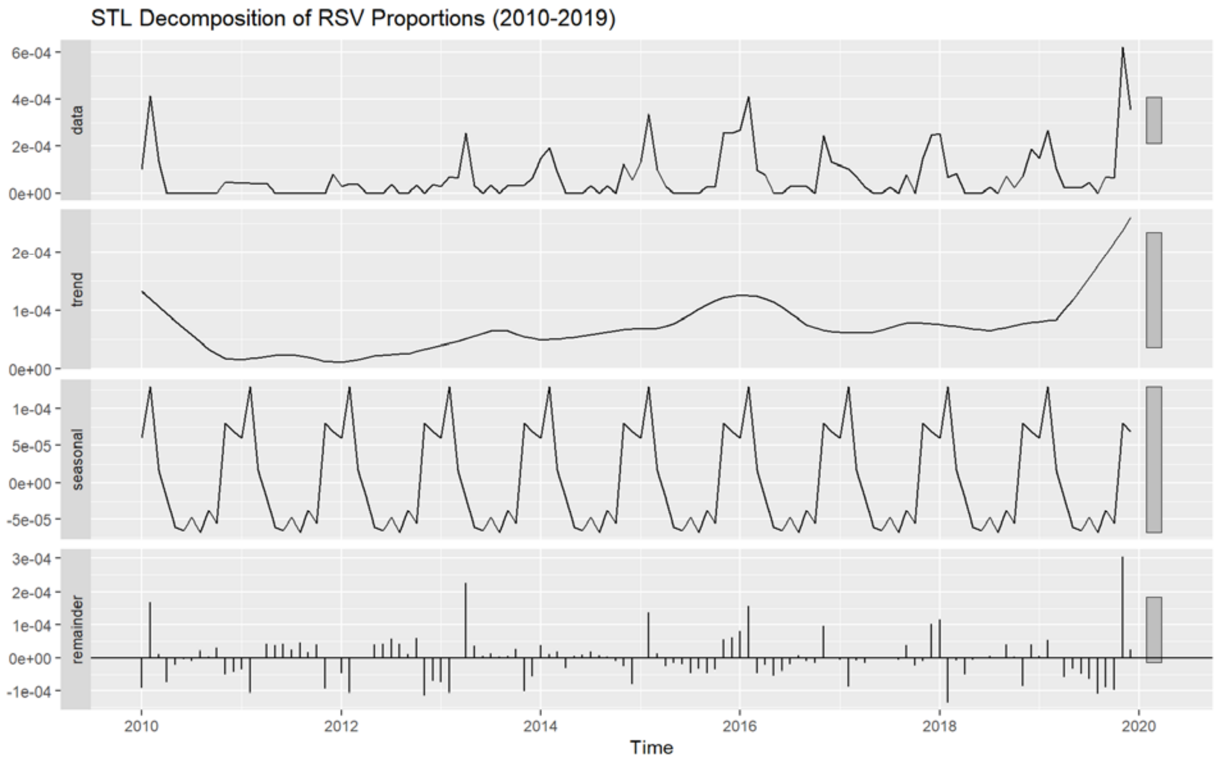

eFigure 3. STL Decomposition of RSV Report Proportions Over Time. The raw time series (top panel) was decomposed into a long-term trend component (second panel), a seasonal component reflecting recurring within-year fluctuations (third panel), and a remainder capturing unexplained variation (bottom panel). Moderate seasonal structure is evident, consistent with a seasonality strength of 0.508 calculated from this decomposition.

medRxiv preprint doi: https://doi.org/10.1101/2025.08.20.25331677; this version posted August 24, 2025. The copyright holder for this preprint (which was not certified by peer review) is the author/funder, who has granted medRxiv a license to display the preprint in perpetuity.
It is made available under a CC-BY 4.0 International license .

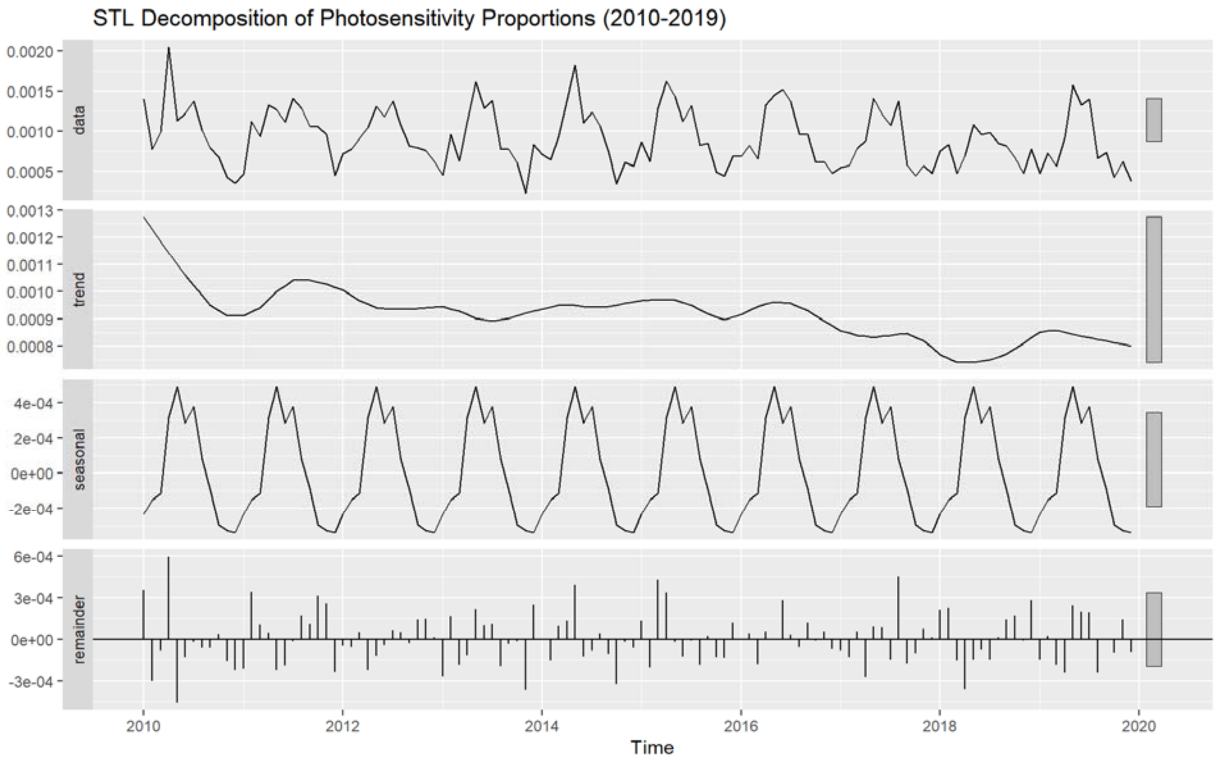

eFigure 4. STL Decomposition of Photosensitivity Report Proportions Over Time. The raw time series (top panel) was decomposed into a long-term trend component (second panel), a seasonal component reflecting recurring within-year fluctuations (third panel), and a remainder capturing unexplained variation (bottom panel). Strong seasonal structure is evident, consistent with high seasonality strength (0.704) calculated from this decomposition.

medRxiv preprint doi: https://doi.org/10.1101/2025.08.20.25331677; this version posted August 24, 2025. The copyright holder for this preprint (which was not certified by peer review) is the author/funder, who has granted medRxiv a license to display the preprint in perpetuity.
It is made available under a CC-BY 4.0 International license .

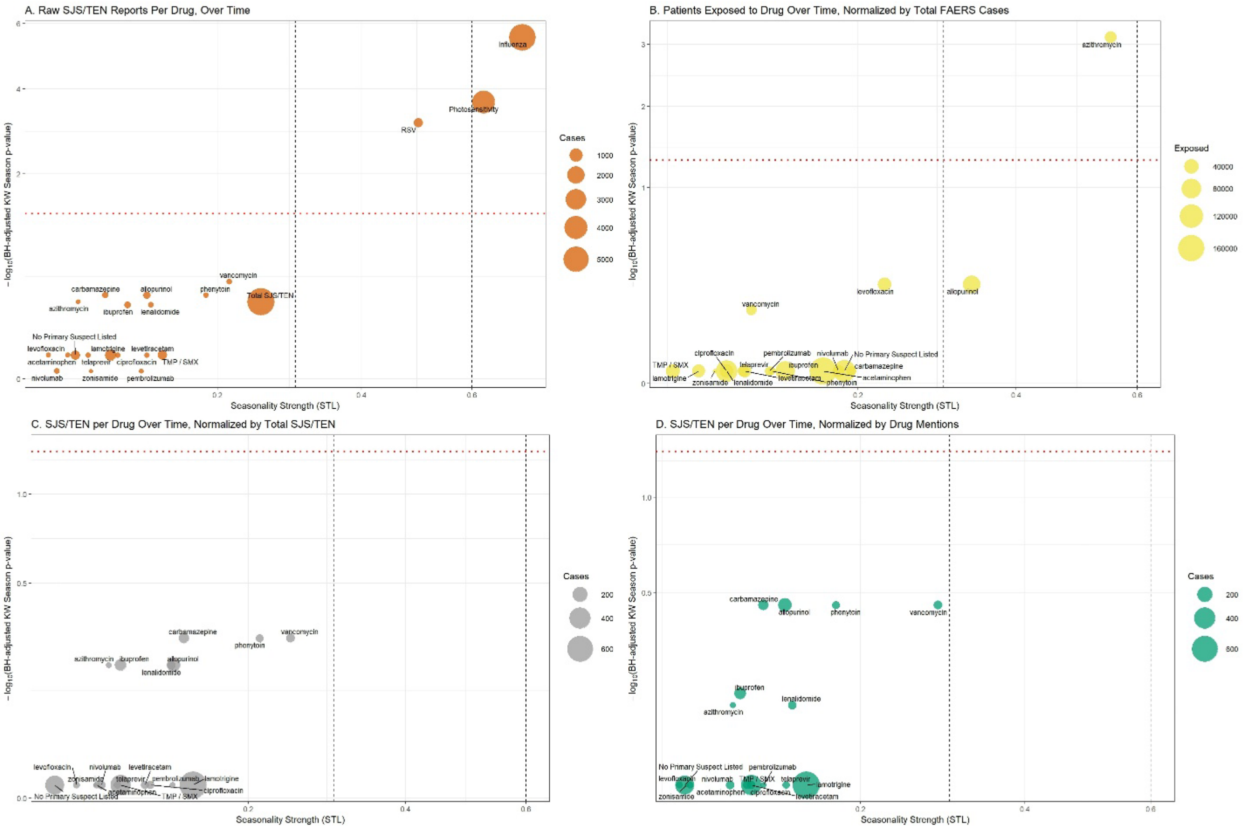

eFigure 5. Seasonality Strength and Statistical Significance of SJS/TEN Reporting Across Drugs Using Multiple Normalization Strategies. Bubble plots showing seasonality strength (x-axis, from STL decomposition) versus statistical significance of seasonal variation (-$\log_{10}$ Benjamini-Hochberg adjusted p-value from Kruskal-Wallis tests; y-axis) for drugs associated with at least 50 SJS/TEN cases in FAERS from 2010–2019. Bubble size reflects the number of cases or exposures. Dashed vertical lines at 0.3 and 0.6 demarcate weak, moderate, and strong seasonality thresholds. The red horizontal line marks the significance threshold (adjusted p = 0.05).

(A) Raw SJS/TEN reports per drug over time.

(B) Number of patients exposed to each drug over time, normalized by total FAERS reports.

(C) SJS/TEN reports per drug, normalized by total SJS/TEN cases.

(D) SJS/TEN reports per drug, normalized by drug mentions (as a proxy for exposure).

medRxiv preprint doi: https://doi.org/10.1101/2025.08.20.25331677; this version posted August 24, 2025. The copyright holder for this preprint (which was not certified by peer review) is the author/funder, who has granted medRxiv a license to display the preprint in perpetuity.
It is made available under a CC-BY 4.0 International license .

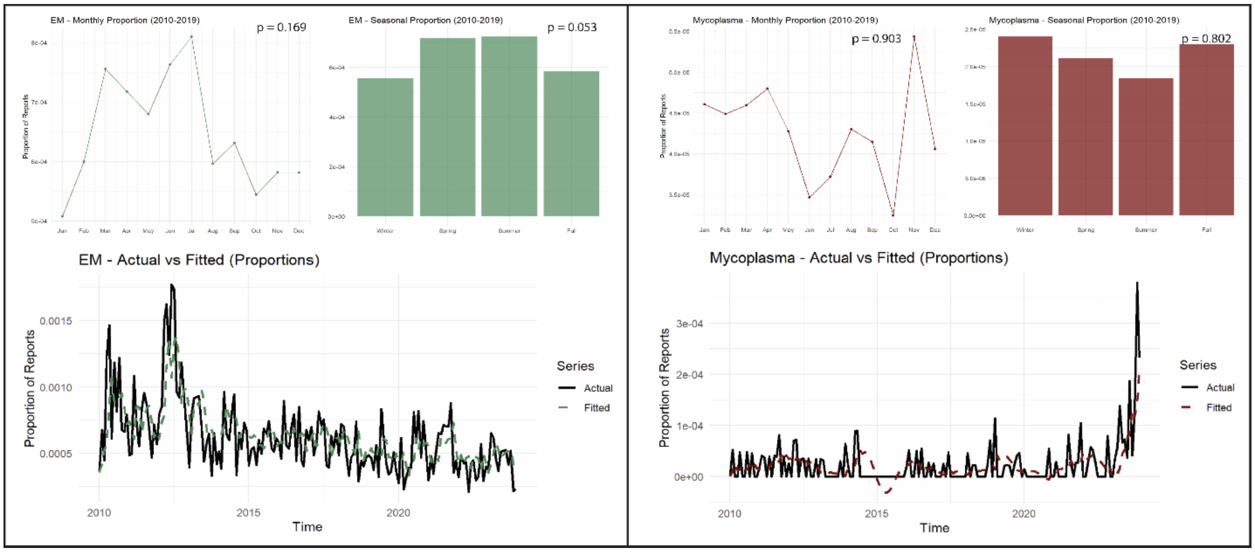

eFigure 6. Respiratory Mycoplasma and EM Summary. Neither EM nor respiratory *Mycoplasma* infection showed statistically significant seasonality by month or by season from 2010-2019, or by STL decomposition (0.256 for EM, 0.177 for *Mycoplasma*). A marked increase in *Mycoplasma* reporting was noted near the end of the study period (2023).

medRxiv preprint doi: https://doi.org/10.1101/2025.08.20.25331677; this version posted August 24, 2025. The copyright holder for this preprint (which was not certified by peer review) is the author/funder, who has granted medRxiv a license to display the preprint in perpetuity.
It is made available under a CC-BY 4.0 International license .

Drug or Condition	Cases	Exposed	Seasonal Strength	Kruskal-Wallis P-value (Seasonal)		Kruskal-Wallis P-value (Monthly)		Incidence	Exposure
				Unadjusted	Adjusted	Unadjusted	Adjusted		
Total SJS/TEN	5265		0.145	9.330×10^{-1}	9.891×10^{-1}	9.380×10^{-1}	9.980×10^{-1}		
Influenza	5247		0.793	6.480×10^{-8}	1.426×10^{-6}	2.420×10^{-16}	5.324×10^{-15}		
Photosensitivity	3634		0.666	4.150×10^{-6}	4.565×10^{-5}	4.820×10^{-12}	5.302×10^{-11}		
lamotrigine	600	26402	0.087	9.862×10^{-1}	9.891×10^{-1}	9.930×10^{-1}	9.980×10^{-1}		
TMP / SMX	336	33046	0.072	9.107×10^{-1}	9.891×10^{-1}	7.468×10^{-1}	9.980×10^{-1}		
RSV	281		0.445	1.650×10^{-4}	1.210×10^{-3}	7.360×10^{-10}	5.397×10^{-9}		
No Primary Suspect Listed	276	97061	0.069	9.550×10^{-1}	9.891×10^{-1}	8.261×10^{-1}	9.980×10^{-1}		
allopurinol	146	54533	0.148	3.609×10^{-2}	1.985×10^{-1}	3.161×10^{-1}	9.935×10^{-1}		
ibuprofen	114	73796	0.083	4.305×10^{-1}	8.610×10^{-1}	5.063×10^{-1}	9.980×10^{-1}		
carbamazepine	85	12323	0.099	1.927×10^{-1}	6.134×10^{-1}	7.201×10^{-1}	9.980×10^{-1}		
lenalidomide	74	96062	0.109	3.031×10^{-1}	7.048×10^{-1}	6.596×10^{-1}	9.980×10^{-1}		
nivolumab	71	21305	0.049	9.376×10^{-1}	9.891×10^{-1}	9.804×10^{-1}	9.980×10^{-1}		
vancomycin	66	15743	0.165	2.172×10^{-1}	6.134×10^{-1}	8.293×10^{-2}	3.649×10^{-1}		
telaprevir	65	21349	0.085	8.321×10^{-1}	9.891×10^{-1}	9.980×10^{-1}	9.980×10^{-1}		
phenytoin	64	7125	0.189	9.288×10^{-2}	4.087×10^{-1}	4.481×10^{-1}	9.980×10^{-1}		
levetiracetam	63	27712	0.128	5.811×10^{-1}	9.891×10^{-1}	4.330×10^{-2}	2.382×10^{-1}		
levofloxacin	59	31435	0.070	8.867×10^{-1}	9.891×10^{-1}	9.776×10^{-1}	9.980×10^{-1}		
azithromycin	58	22025	0.101	3.204×10^{-1}	7.048×10^{-1}	3.877×10^{-1}	9.980×10^{-1}		
acetaminophen	51	158744	0.060	5.907×10^{-1}	9.891×10^{-1}	7.700×10^{-1}	9.980×10^{-1}		
ciprofloxacin	51	30727	0.129	2.231×10^{-1}	6.134×10^{-1}	2.063×10^{-1}	7.565×10^{-1}		
zonisamide	51	3743	0.065	9.665×10^{-1}	9.891×10^{-1}	8.039×10^{-1}	9.980×10^{-1}		
pembrolizumab	50	10486	0.131	9.891×10^{-1}	9.891×10^{-1}	9.709×10^{-1}	9.980×10^{-1}		

eTable 1. Seasonality Measures – Northern Hemisphere (Restricted)

medRxiv preprint doi: https://doi.org/10.1101/2025.08.20.25331677; this version posted August 24, 2025. The copyright holder for this preprint (which was not certified by peer review) is the author/funder, who has granted medRxiv a license to display the preprint in perpetuity.
It is made available under a CC-BY 4.0 International license .

Drug or Condition	Cases	Exposed	Seasonal Strength	Kruskal-Wallis P-value (Seasonal)		Kruskal-Wallis P-value (Monthly)		Incidence	Exposure
				Unadjusted	Adjusted	Unadjusted	Adjusted		
Influenza	2722		0.600	2.940×10^{-7}	2.646×10^{-6}	1.500×10^{-13}	1.350×10^{-12}	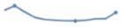	
Photosensitivity	2216		0.633	2.160×10^{-5}	9.720×10^{-5}	1.050×10^{-10}	4.725×10^{-10}	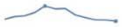	
Total SJS/TEN	1832		0.089	9.780×10^{-1}	9.780×10^{-1}	9.910×10^{-1}	9.910×10^{-1}	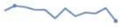	
RSV	221		0.431	8.370×10^{-4}	2.511×10^{-3}	3.080×10^{-8}	9.240×10^{-8}	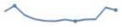	
lamotrigine	175	16331	0.178	1.135×10^{-1}	2.553×10^{-1}	1.784×10^{-1}	4.013×10^{-1}	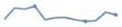	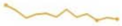
TMP / SMX	171	14578	0.134	8.120×10^{-1}	9.135×10^{-1}	5.790×10^{-1}	9.910×10^{-1}	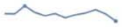	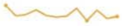
No Primary Suspect Listed	124	67926	0.079	5.853×10^{-1}	9.020×10^{-1}	6.699×10^{-1}	9.910×10^{-1}	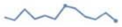	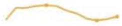
allopurinol	61	26224	0.075	7.016×10^{-1}	9.020×10^{-1}	9.088×10^{-1}	9.910×10^{-1}	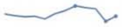	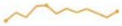
lenalidomide	50	79867	0.057	6.781×10^{-1}	9.020×10^{-1}	8.932×10^{-1}	9.910×10^{-1}	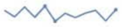	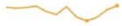

eTable 2. Seasonality Measures – US Only

medRxiv preprint doi: https://doi.org/10.1101/2025.08.20.25331677; this version posted August 24, 2025. The copyright holder for this preprint (which was not certified by peer review) is the author/funder, who has granted medRxiv a license to display the preprint in perpetuity.
It is made available under a CC-BY 4.0 International license .

Forecast Error Metrics and Model Diagnostics

Outcome	MSE (Pre-COVID)	RMSE (Pre-COVID)	MSE (Post-COVID)	RMSE (Post-COVID)	ARIMA Model	Model Coefficients	Ljung-Box Test
SJS/TEN	1.05e-07	3.24e-04	2.52e-07	5.02e-04	ARIMA(0,1,1) with drift	ma1 = -0.871<br>drift = -1.16e-05	Q* = 30.3<br>df = 23<br>p = 0.141
Influenza	3.53e-07	5.94e-04	2.82e-06	1.68e-03	ARIMA(1,0,0)(2,1,2)[12]	ar1 = 0.489<br>sar1 = 0.698<br>sar2 = -0.479<br>sma1 = -1.42<br>sma2 = 0.759	Q* = 14<br>df = 19<br>p = 0.782
RSV	7.17e-09	8.47e-05	3.36e-08	1.83e-04	ARIMA(3,1,2)	ar1 = 1.17<br>ar2 = -0.116<br>ar3 = -0.467<br>ma1 = -1.75<br>ma2 = 0.885	Q* = 26.9<br>df = 19<br>p = 0.107
Photosensitivity	4.50e-08	2.12e-04	4.69e-08	2.17e-04	ARIMA(0,0,0)(0,1,1)[12] with drift	sma1 = -0.879<br>drift = -2.07e-06	Q* = 23.7<br>df = 23<br>p = 0.423

eTable 3. Time Series and Forecasting Accuracy
